# SARS-CoV-2-Related Kidney Injury: Current Concern and Challenges

**DOI:** 10.1007/s42399-020-00529-0

**Published:** 2020-09-23

**Authors:** Yongqian Cheng, Wenling Wang, Liang Wu, Guangyan Cai

**Affiliations:** 1 Department of Geriatrics, The Fifth Medical Center of Chinese PLA General Hospital, Beijing, 100039 China; 2 Department of Nephrology, The First Medical Center of Chinese PLA General Hospital, National Clinical Research Center for Kidney Diseases, State Key Laboratory of Kidney Diseases, Beijing Key Laboratory of Kidney Disease Research, Chinese PLA Institute of Nephrology, Beijing, 100853 China

**Keywords:** Coronavirus disease 2019, Chronic kidney disease, Kidney injury, Pathogenesis, Treatment

## Abstract

Coronavirus disease 2019 (COVID-19) not only causes pulmonary inflammation but also causes multiple organ damages, including the kidney. ACE2, as one of the receptors for SARS-CoV-2 intrusion, is widely distributed in kidney tissues. Currently, the diagnosis and treatment of SARS-CoV-2 infection in patients with chronic kidney disease (CKD) are still unclear. Here, we review the recent findings of characteristics of COVID-19 in CKD patients and highlight the possible mechanisms of kidney injury caused by SARS-CoV-2 infection. We then discuss the emerging therapeutic approaches aimed at reducing kidney damage and protecting kidney function including virus removal, immunotherapy, supporting treatment, special blood purification therapy, etc. Problems unresolved and challenges ahead are also discussed.

## Introduction

Coronavirus disease 2019 (COVID-19) is the first confirmed pandemic sparked by a coronavirus in the twenty-first century. As COVID-19 continues to spread globally, inevitably, it may trigger new challenges and issue to patients with confounding chronic diseases. Many studies have confirmed that comorbidities are important risk factors for the severity and outcome of SARS-CoV-2 infection [1]. Chronic kidney disease (CKD) [2] is not uncommon and represents one of the chronic diseases witnessed during COVID-19 ranging from 8.5 to 9.8% so far reported [3]. In this article, we reviewed the impact of SARS-CoV-2 infection in disease progression and outcome in CKD patients and relevant interests or topics.

## Does CKD Aggravate the Condition of Patients with COVID-19?

It was reported that 20–51% of hospitalized patients have one or more comorbidities. The most prevailing ones are diabetes mellitus (8.2–20%), hypertension (9.5–31.2%), and other cardiovascular and cerebrovascular diseases (5.6–40%) [1, 4–7]. The prevalence of CKD was 0–5.6%, which represents a minor proportion in COVID-19 patients [6, 8–14] (Table 1).

**Table 1:** Prevalence of number of main comorbidities in COVID-19 patients in each comparative study

Study	Subtype	Participants		Age median (IQR)/mean	Comorbidities (*n*, %)								
		Total	Male (*n*, %)		Any comorbidity	CKD	Diabetes	Hypertension	COPD	Cardiovascular disease	Cerebrovascular disease	Malignancy	Chronic liver disease
Guan et al. [8]	Severe	173	100 (57.8)	52.0 (40.0–65.0)	67 (38.7)	3 (1.7)	26 (16.2)	41 (23.7)	6 (3.5)	10 (5.8)	4 (2.3)	3 (1.7)	1 (0.6)
	Non-severe	926	540 (58.2)	45.0 (34.0–57.0)	194 (21.0)	5 (0.5)	53 (5.7)	124 (13.4)	6 (0.6)	17 (1.8)	11 (1.2)	7 (0.8)	22 (2.4)
Wang et al. [6]	Severe	36	22 (61.1)	66.0 (57.0–78.0)	26 (72.2)	2 (5.6)	8 (22.2)	21 (58.3)	3 (8.3)	9 (25.0)	6 (16.7)	4 (11.1)	0 (0.0)
	Non-severe	102	53 (52.0)	51.0 (37.0–62.0)	38 (37.3)	2 (2.0)	6 (5.9)	22 (21.6)	1 (1.0)	11 (10.8)	1 (1.0)	6 (5.9)	4 (3.9)
Zhang et al. [9]	Severe	58	33 (56.9)	64.0 (25.0–87.0)	46 (79.3)	2 (3.4)	8 (13.8)	22 (37.9)	2 (3.4)	4 (6.9)	2 (3.4)	NR	NR
	Non-severe	82	38 (46.3)	51.5 (26.0–78.0)	44 (53.7)	0 (0.0)	9 (11.0)	20 (24.4)	0 (0.0)	3 (3.7)	1 (1.2)	NR	NR
Wu et al. [10]	Severe	83	45 (54.2)	63.0 (10.2)	NR	2 (2.4)	NR	NR	1 (1.2)	43 (51.8)*		2 (2.4)	4 (4.8)
	Non-severe	197	106 (53.8)	37.6 (17.1)	NR	1 (0.5)	NR	NR	0 (0.0)	14 (7.1)*		3 (1.5)	3 (1.5)
Fang et al. [11]	Severe	24	18 (75.0)	56.7 (14.4)	NR	0 (0.0)	4 (16.7)	11 (45.8)	0 (0.0)	2 (8.3)	3 (12.5)	0 (0.0)	1 (4.2)
	Non-severe	55	27 (49.1)	39.9 (14.9)	NR	3 (5.5)	4 (7.3)	5 (9.1)	0 (0.0)	1 (1.8)	0 (0.0)	1 (1.8)	2 (3.6)
Xiong et al. [12]	Severe	31	NR	NR	NR	1 (3.2)	6 (19.4)	10 (32.3)	4 (12.9)	NR	2 (6.5)	3 (9.7)	1 (3.2)
	Non-severe	58	NR	NR	NR	2 (3.4)	8 (13.8)	16 (27.6)	1 (1.7)	NR	4 (6.9)	8 (13.8)	0 (0)
Zhou et al. [13]	Survivor	137	81 (59.0)	52.0 (45.0–58.0)	55 (40.0)	0 (0.0)	19 (14.0)	32 (23.0)	2 (1.0)	2 (1.0)	NR	2 (1.0)	NR
	Non-survivor	54	38 (70.0)	69.0 (63.0–76.0)	36 (70.0)	2 (4.0)	17 (31.0)	26 (48.0)	4 (7.0)	13 (24.0)	NR	0 (0.0)	NR
Chen et al. [14]	Severe	25	NR	60 (41.8–68)	20 (80.0)	1 (4.0)	4 (16)	11 (44.0)	2 (8.0)	4 (16.0)	2 (8.0)	NR	NR
	Non-severe	23	NR	49 (4.0–63.9)	3 (13.0)	0 (0.0)	0 (0.0)	1 (4.3)	0 (0.0)	0 (0.0)	1 (4.3)	NR	NR
Chen et al. [14]	Survivor	48	NR	NR	23 (47.9)	1 (2.0)	4 (8.3)	12 (25.0)	2 (4.2)	4 (8.3)	3 (6.2)	NR	NR
	Non-survivor	6	NR	70.0 (69.3–77.5)	4 (66.7)	0 (0.0)	0 (0.0)	1 (16.7)	1 (16.6)	2 (33.3)	0 (0.0)	NR	NR

*Means cardiovascular disease and cerebrovascular disease

*n* number, *NR* not reported, *CKD* chronic kidney disease, *COPD* chronic obstructive pulmonary disease

A national wide analysis demonstrated that 25.1% (399/1590) of COVID-19-infected patients may be confounded by at least one comorbidity, which significantly differed from patients without any, in risk of worse clinical outcomes. The hazard ratio (HR) among patients with two or more comorbidities was greater than those with one comorbidity [2.59 (95% CI 1.61–4.17) vs. 1.79 (95% CI 1.16–2.77)] [1]. Concordantly, previous studies also demonstrated that the number of comorbidities was correlated with severity and accumulative clinical outcomes [1].

Although the primarily targeted organs of COVID-19 include but not limited to the respiratory, immune, and coagulation systems, CKD may represent an additional risk factor for augmented impairment of these systems. The reported morbidity of CKD in severe COVID-19 patients is even higher than non-severe patients (1.7–5.6% vs. 0–3.6%) [6, 8–14] (Table 1), consistent with observed higher incidence in patients admitted to the intensive care unit (ICU) comparing with non-ICU patients (2/36(5.6%) vs. 2/102 (2.0%) [6]. A meta-analysis including 1389 COVID-19 patients showed the significant association between CKD and severe COVID-19. When data of individual studies were pooled, there was no relevant heterogeneity [odds ratio (OR) 3.03 (95% CI 1.09–8.47), *I*^2^ = 0.0%, Cochran’s Q, *p* = 0.84]. As a result, CKD seems to be associated with enhanced risk of severe COVID-19 infection [15]. Oyelade et al. found an increased risk of severity and mortality in COVID-19 patients with CKD [16]. In patients with COVID-19 and CKD, 83.93% (47/56) of cases were severe and 53.33% (8/15) mortality was also reported. Albeit in the other direction, there were little data perceiving to the outbreak incidence of COVID-19 in CKD cohort, comparing with the general population.

## Does SARS-CoV-2 Aggravate Kidney Damage in CKD Patients?

Some studies about renal function in COVID-19 patients have shown common manifestations of kidney dysfunctions and incidental acute kidney injury [3, 17]. Clinically, the incidence of acute kidney injury in COVID-19 varied from 0 to 66% in different centers [17, 18]. There were 59%, 44%, 14%, and 10% patients who had features of proteinuria, hematuria, and elevated blood urea nitrogen or serum creatinine, respectively. According to the recent studies, 10–14.4% of COVID-19 patients were found with increased creatinine or urea nitrogen, 7.2–59% patients exhibited proteinuria, and 26.7–44% patients had hematuria [17–19] (Table 2). In total, 27.06% (23/85) patients for first time concurred with AKI after infecting SARS-CoV-2 [20]. Over 40% of patients infected with SARS-CoV-2 had prior evidence of kidney diseases, and the presence of kidney diseases was associated with increased risk of mortality during hospitalization [19]. Risk of mortality in COVID-19 patients with AKI was 5.3 folders of that without AKI. The comorbidities of AKI were 37.5% in non-survivors [21]. The incidence of AKI was 66% in severe patients [17], and it was higher in patients with elevated baseline serum creatinine compared with normal baseline serum creatinine (11.9% vs. 4.0%) CKD [19]. It is worth notification that patients under SARS-CoV-2 infection, especially severe one, have varied degrees of kidney injury. In CKD patients, the renal impairment was further aggravated, and AKI surged significantly higher than those without CKD.

**Table 2:** Renal test abnormalities from various COVID-19 studies

Study		Number	Age (median or mean)	Serum creatinine increased	Blood urea nitrogen increased	Proteinuria	Hematuria	AKI
Cheng et al. [19]	Total	701	63	14.4%	13.1%	43.9%	26.7%	5.1%
	Elevated baseline Scr	101	73	–	–	69.8%	52.8%	11.9%
	Normal baseline Scr	600	61	–	–	40.4%	23.1%	4%
Li et al. [17]	Total	193	57	10%	14%	59%	44%	28%
	Severe	65	66	20%	29%	65%	45%	66%
	Non-severe	128	55	5%	6%	55%	44%	9%
Wang et al. [18]	Total	116	54	10.8%*		7.2%	NA	0%

*Means serum creatinine or blood urea nitrogen increased

*Scr* serum creatinine

Controversial results also exist like another study [18] indicating that SARS-CoV-2 infection was not found significantly correlated with incremental acute renal injury or aggravate chronic kidney failure in the COVID-19 patients. In that study, 10.8% of patients showed mild elevation of blood urea nitrogen or creatinine, while 7.2% with trace amount or 1+ albuminuria.

In the current study, kidney injury in patients with COVID-19 is mainly evaluated based on serum creatinine elevation. So far, limited studies were available in prospective design by incorporating more sensitive surrogate markers of early-onset kidney injury, such as urinary β-2 microglobulin, microalbumin, NAGL, etc. Furthermore, it is also critical to understand prognosis of COVID-19 patients with kidney injury or even AKI, especially in those with predisposed CKD.

In order to evaluate the risk of serious adverse outcomes in COVID-19 patients according to the number and type of comorbidities, Guan et al. [1] have analyzed 1590 laboratory-confirmed hospitalized patients in 31 province/autonomous regions/provincial municipalities from mainland China. 25.1% (399/1590) was reported to have at least one comorbidity. 8.2% (131/1590) of patients reached the composite endpoints, which consisted of admission to intensive care unit, or invasive ventilation, or death, and 8.2% (130/1590) had two or more comorbidities. The HR was 1.79 (95% CI 1.16–2.77) vs. 2.59 (95% CI 1.61–4.17) among patients with at least one comorbidity or two or more comorbidities. The prevalence of CKD was 1.3% (21/1590). It is the lowest one compared with hypertension (269; 16.9%), diabetes (130; 8.2%), other cardiovascular diseases (59; 3.7%), cerebrovascular diseases (30; 1.9%), hepatitis B infections (28; 1.8%), or chronic obstructive pulmonary disease (COPD) (24; 1.5%). Intriguingly, significantly more patients with CKD (28.6% vs. 8.0%) reached the composite endpoints compared with those without, which ranked behind COPD (50.0% vs. 7.6%), malignancy (38.9% vs. 7.9%), and cerebrovascular diseases (33.3% vs. 7.8%), albeit higher than diabetes (23.8% vs. 6.8%), cardiovascular diseases (22.0% vs. 7.7%), and hypertension (19.7% vs. 5.9%). It is herein postulated that COVID-19 patients with underlying CKD are at higher risk of progression to severe conditions.

Lab test findings in the CKD patients with COVID-19 infection showed that the leukocyte count in patients with elevated baseline serum creatinine was 9.5 ± 8.0 × 10^9^/L, which was significantly higher than those with normal baseline serum creatine (7.2 ± 7.4 × 10^9^/L). While the lymphocyte count and platelet count in the patients with elevated baseline serum creatinine was significantly lower than those in the patients with normal serum creatinine (0.8 ± 0.5 × 10^9^/L vs. 0.9 ± 0.5 × 10^9^/L, 191 ± 94 × 10^9^/L vs. 216 ± 94 × 10^9^/L) [19].

Radiologic finding on chest computed tomography (CT) showed that 86.2% patients with COVID-19 revealed abnormal results. The most common patterns on chest CT were ground-glass opacity (56.4%) and bilateral patchy shadowing (51.8%) [8]. In COVID-19 patients with CKD, abnormal chest X-ray and chest CT were showed in 15.3% and 71.1% patients, respectively, while in non-CKD COVID-19 patients, it was 14.3% and 66.7%, respectively, CT [1]. In HD patients with COVID-19, 41% of patients showed unilateral infiltrates, 59% showed bilateral infiltrates, and 62% had multiple ground-glass opacity lesions [22], while in kidney transplant patients with COVID-19, 85% patients showed infiltrate chest X-ray at hospital admission. Chest radiographs were repeated in 15/20 patients, and radiological findings worsened in 87% 13/15 patients [23]. A recent study by LI et al. showed that the median kidney CT value of 110 patients with COVID-19 was 27.3 HU which was significantly lower than the healthy control group who had no kidney diseases (*n* = 109, 33.2 HU) and the patients with other pneumonia (*n* = 28, 32.8 HU). It indicated that inflammation and edema of the renal parenchyma may commonly occur in COVID-19 patients [17]. Unfortunately, there was no statistical comparison data about the imaging of the lungs or kidneys in patients with CKD or not.

### COVID-19 in Hemodialysis Patients

Hemodialysis [3] patients are another special population in CKD. In the study conducted by Ma et al. [22], there were 37 (16.09%) HD patients diagnosed positive for COVID-19 with 6 among them perished. In that study, the clinical symptoms such as fever, fatigue, cough, chest pain, and nausea were not prominent in HD patients with COVID-19. Compared with non-HD patients with COVID-19, the proportion of T cell counting and plasma cytokines such as IL-4, IL-6, IL-10, TNF-α, and INF-γ was lower in HD patients infected by SARS-CoV-2. A case report by Tang et al. [24] also mentioned profile of one HD patient infected with SARS-CoV-2. This patient displayed a relatively mild course despite multiple comorbid conditions including HBV infection and diabetes. Wang et al. reported that up to the end of their study, none of the 5 HD patients with COVID-19 infection had developed acute respiratory distress syndrome, shock, or other serious complications [25].

From evidence aforementioned, it could be stipulated that HD patients may be vulnerable to SARS-CoV-2 due to their impaired immune system, but in parallel, they may not suffer from the worst condition due to less triggered immune response-mediated pathogenesis as symbolized by low cytokines in plasma. More studies are warranted to investigate and provide solid evidences.

### COVID-19 in Kidney Transplant Recipients

Kidney transplant recipients are being treated with long-term immunosuppression agents. The outcome of SARS-CoV-2 infection in these patients remains unclear. Banerjee et al. [26] reported on seven cases of COVID-19 in kidney transplant recipients, all displayed respiratory symptoms and fever. Dosage of immunosuppressant was modified in six of seven patients. Four was transferred to the ICU, and one patient died after 12 days from symptom onset.

In another recent study with 20 kidney transplant recipients, SARS-CoV-2-induced pneumonia was characterized by high risk of deterioration and significant mortality [23]. In this study, twenty kidney transplant recipients with median post-transplant 13 years were enrolled. All had immunosuppressant discontinued and were started on methylprednisolone. They experienced a rapid clinical deterioration and escalating oxygen requirement. Six patients developed AKI with one requiring HD, and among them five patients died after a median period of 15 days from symptom onset. Therefore, SARS-CoV-2 infection in kidney transplant patients from this study showed that such cases may be severe enough requiring intensive care admission and these patients are in high risk of disease progression and death.

## Possible Mechanisms of Kidney Injury in Patients Infected with SARS-CoV-2

### Direct Mechanism of Action of SARS-CoV-2 to Kidney Injury

#### Deposition of Molecules Associated with SARS-CoV-2 Infection in Renal Tissues

Viral attachment, fusion, and subsequent replication of coronavirus in targeted host cells are a complex process which remains elusive. However, it has been revealed that there required one synergistic action of S1 protein binding to receptors on the cell surface and a series of proteolysis when virus attached to the cell surface. Researchers have found that at least three proteins are indispensably required in this process, including ACE2, type II transmembrane serine proteases (TMPRSS2), and an enzyme called furin. Surprisingly, these molecules were found to be abundant in kidney tissue which indicates that kidney tissue might be target cells invaded by SARS-CoV-2.

#### Angiotensin-Converting Enzyme 2

ACE2 belongs to the angiotensin-converting enzyme family of dipeptidyl carboxydipeptidases and is homologous to human angiotensin-1-converting enzyme. It had been illustrated that ACE2 was one of the major receptors which mediated the entry of SARS-CoV-2 into human cells. A study by Fan and colleagues [27] showed that ACE2 mRNA level and ACE2 protein are both higher in kidney cells. The expression distribution of ACE2 suggests that a potential mechanism of infection and direct damage of kidney may be caused by SARS-CoV-2 binding to ACE2. Another study based on single-cell analysis by Lin and colleagues [28] also found that ACE2 was enriched in proximal tubular cells which may indicate that the kidney is more susceptible to SARS-CoV-2 infection.

Previous studies have confirmed that ACE2 exists in a variety of renal tissue cells. The expression of ACE2 was strongly positive in the apical border of the proximal tubular cells whereas less present in cytoplasm. Glomerular visceral was weak ACE2 staining, and the mesangium and glomerular endothelium were negative [29]. Recently, Caibin Fan et al. searched literatures and analyzed the online datasets showing that the expression level of ACE2 protein is significantly higher in the kidney, especially in renal tubular cells [27]. It could be speculated that SARS-CoV-2 may directly impair the kidney via proximal renal tubular injury.

#### Furin-Like Cleavage Site

Previous study identified that a peculiar “RRAR motif” in SARS-CoV-2 is a furin recognition site eligible to be activated by an enzyme called furin [30]. Presence of the motif containing this cleavage site between S1/S2 proteins is one of the most critical differences between SARS-CoV and SARS-CoV-2 [30]. Furin is one of the proprotein convertases that can cleave viral envelope glycoproteins specifically, thereby enhancing viral fusion into host cell membranes. Furin had high expression level in the salivary gland, lachrymal gland, colon, liver, and also kidney [31]. Heretofore, SARS-CoV-2 might utilize this specific feature by increasing furin-mediated cleavage site to become more infectious in the kidney and increased kidney injury.

#### Transmembrane Protease Serine 2

The SARS-CoV-2 gains entry to a cell by binding of the spike (S) proteins to ACE2 and S protein priming by host cell proteases. A study by Hoffmann and colleagues [32] demonstrated that the ACE2 receptor was the cellular receptor of SARS-CoV-2 and TMPRSS2 was cell proteases priming of S protein, and ACE2 and TMPRSS2 are both the key point to SARS-CoV-2 infecting human cells. ACE2 receptor and TMPRSS2 both exist in the kidney [27, 33]. That means that the virus has the potential to attack the kidney by this way. Therefore, a TMPRSS2 inhibitor might be useful for antiviral intervention including protecting kidney.

#### Evidence of Direct Virus Infection in Renal Tissue

It was reported that nucleocapsid protein of SARS-CoV-2 was detected in urine in 73.6% of diagnosed COVID-19 patients [34], and SARS-CoV-2 nucleocapsid protein was detected in kidney tissues from postmortem samples [20]. Immunohistochemistry results showed that SARS-CoV-2 NP expression was typically restricted to kidney tubular cells. Recently, Hua Su et al. [35] reported their renal histopathological findings of 26 postmortem COVID-19 patients. Diffuse proximal tubular injury with disarrangement of brush border was observed under light contrast microscope. Clusters of coronavirus particles with distinctive spikes were detectable under electron microscopic examination in the tubular epithelium and podocytes. Single-cell RNA sequencing technology provided the cellular evidence that SARS-Cov-2 invaded human kidney tissue via proximal convoluted tubule, proximal tubule, proximal straight tubule cells, and glomerular parietal cells by means of ACE2-related pathway and used their cellular protease TMPRSS2 for priming [36]. These findings demonstrated direct evidence of the invasion of SARS-CoV-2 into kidney tissue. It was also confirmed that the renal target cells attacked by the SARS-CoV-2 are proximal renal tubular cells, which is consistent with the distribution of ACE2 in renal tissue [37]. They also found an upregulation of ACE2 expression on the surface of renal tubular cells.

### Cytokine Storm and Kidney Injury

Cytokine storm is also an important factor leading to multiple organ damage in COVID-19 patients. High levels of proinflammatory cytokines may lead to shock and tissue damage in the heart, liver, and kidney, as well as respiratory failure or multiple organ failure [35]. Both ICU patients and non-ICU patients had a higher level of IL1B, IL1RA, IL7, IL8, IL9, IL10, FGF, GCSF, GMCSF, IFNγ, IP10, MCP1, MIP1A, MIP1B, PDGF, TNFα, and VEGF than healthy adults [4]. Compared with non-ICU patients, ICU patients had an even higher level of IL2, IL7, IL10, GCSF, IP10, MCP1, MIP1A, and TNFα. Most patients with severe COVID-19 exhibited significantly elevated levels of serum proinflammatory cytokines, including IL-6 and IL-1β as well as IL-2, IL-8, IL-17, G-CSF, GM-CSF, IP10, MCP1, MIP1-α (also known as CCL3), and TNF. In addition, C-reactive protein and D-dimer were found to be abnormally high. Acute kidney injury occurred in 23% of ICU patients, while in non-ICU patients, acute kidney injury seldom occurs [4]. That indicated that kidney injury might be associated with cytokine storm and further studies are needed to investigate the association between inflammatory storm and kidney injury.

**Fig. 1:**
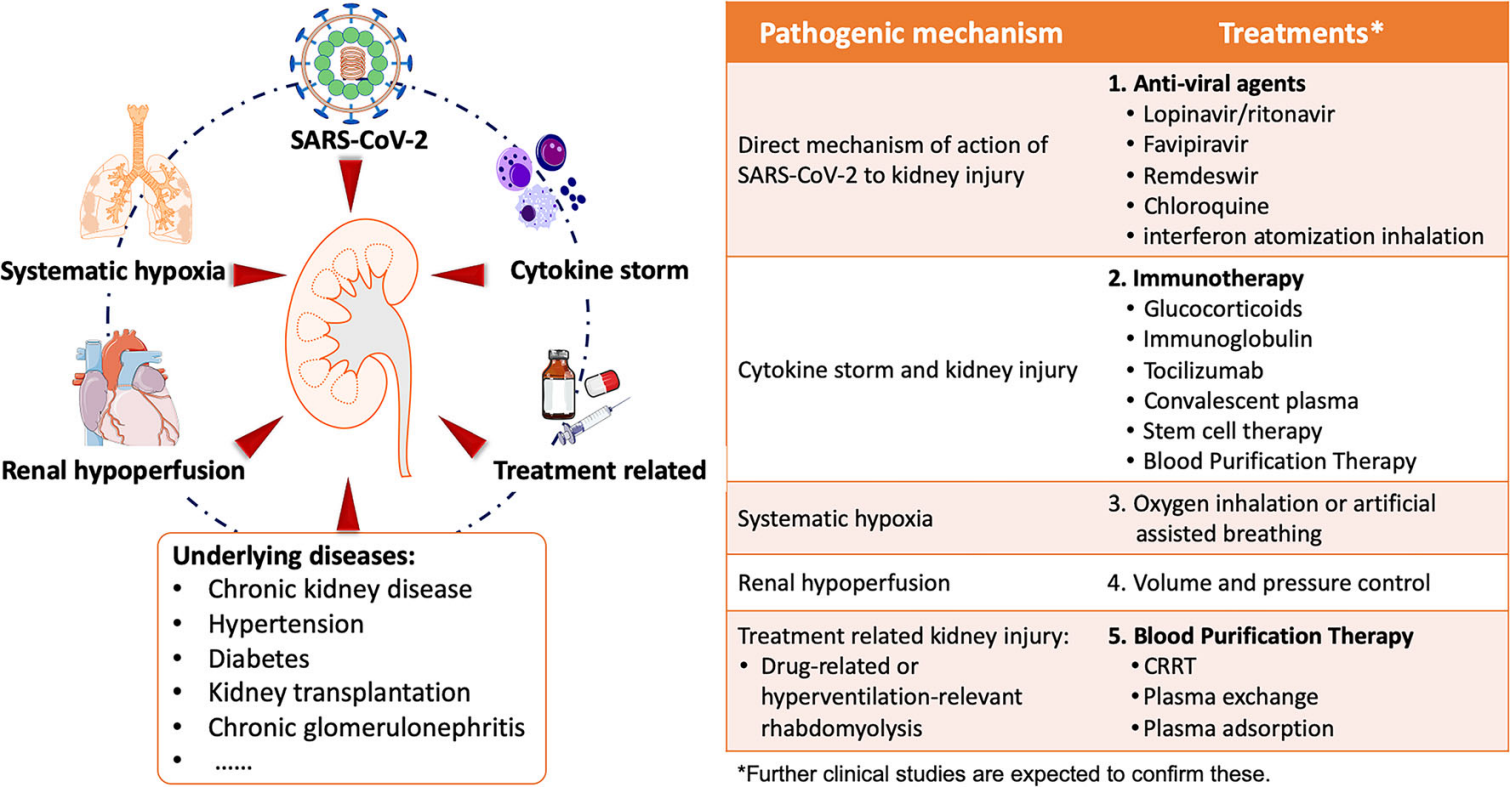
Related factors and possible treatment of COVID-19-related kidney injury

**Fig. 2:**
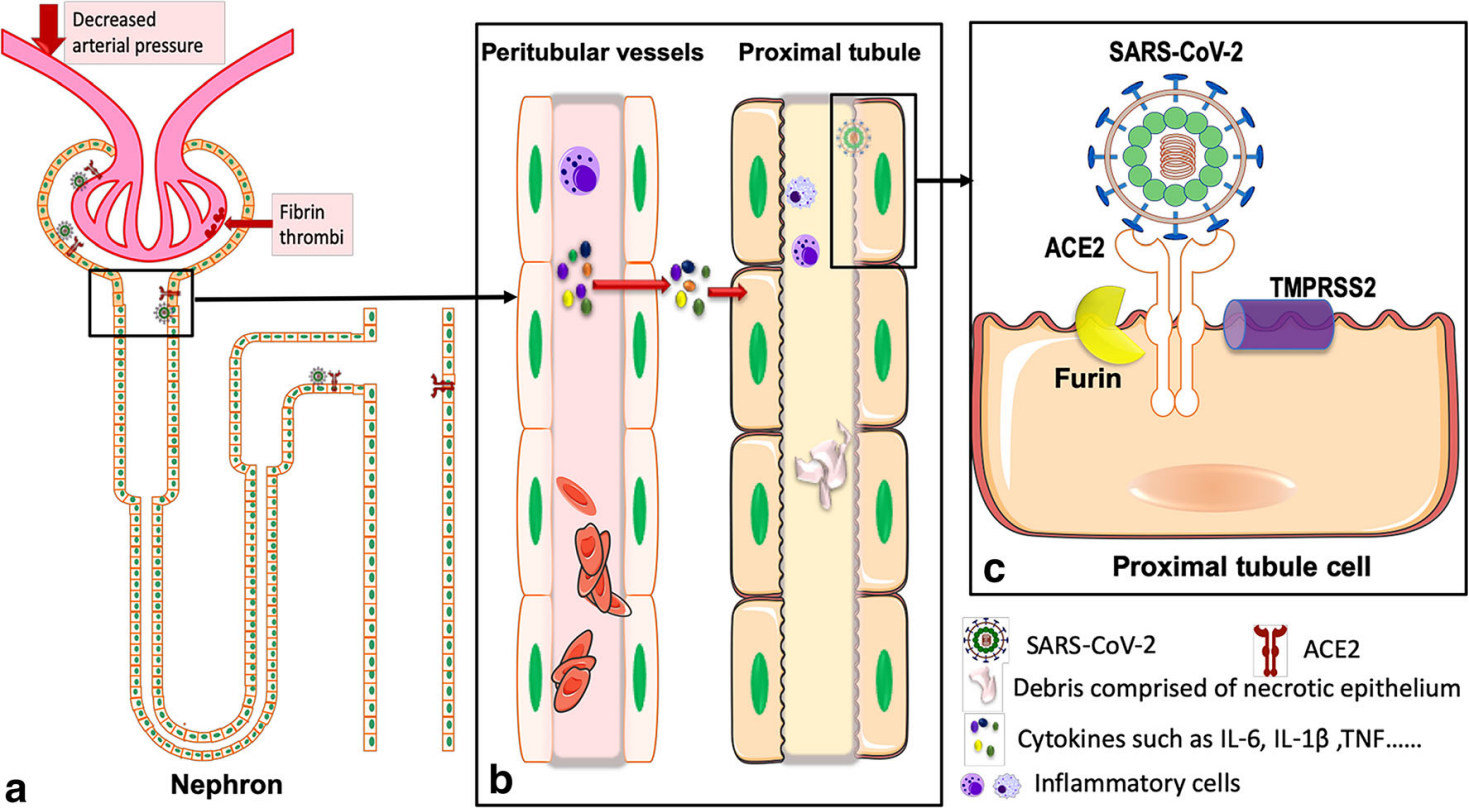
Possible pathogenic mechanisms related with kidney injury. **a** Schematic diagram of the distribution of SARS-CoV-2 in renal tissue confirmed by current studies. SARS-CoV-2 virus particles were detected in cytoplasm of proximal and distal tubular epithelium, as well as in podocytes [35]. ACE2 is mainly distributed in the glomerular visceral and parietal epithelium, in the brush border and cytoplasm of proximal tubular cells, and in the cytoplasm of distal tubules and collecting ducts [29]. Decreased pressure of the glomerular artery and direct viral damage may be associated with glomerular lesions. The main pathological findings were fibrin thrombi and ischemic glomerular contraction [35]. **b** Schematic diagram of pathological damage of proximal convoluted tubules and peritubular capillaries of the kidney. Inflammatory cell infiltration, release of inflammatory cytokines, chemokine, and others caused renal tubular injury. Erythrocyte aggregates obstructed peritubular capillaries, and debris comprised of necrotic epithelium accumulated in tubular lumens [35]. **c** SARS-CoV-2 invades renal tubule-associated molecules. Schematic diagram of proteins associated with SARS-CoV-2 invasion into proximal tubule cell

In addition to the direct virulence of SARS-CoV-2 and cytokine storm, there are potentially other factors attributing to acute kidney injury, such as systematic hypoxia, abnormal coagulation, and possible drug-related or hyperventilation-relevant rhabdomyolysis. All these factors may lead to further impairment of renal function in CKD patients (Figs. 1 and 2).

## Treatment of COVID-19 Patients with CKD

It was believed that not only the virus but also the overactive immune response made people severely ill or cause death. Therefore, the treatment target is antiviral and immunosuppressive. Unfortunately, there are no approved specific antiviral agents targeting the SARS-CoV-2, although there are some promising antiviral drugs in clinical trials, including remdesivir, lopinavir/ritonavir, chloroquine, and hydroxychloroquine. There are also many therapeutic methods in the field of immune regulation for severe COVID-19 patients, including tocilizumab, convalescent plasma (CP) and stem cell therapy, etc., but only glucocorticoid is considered in clinical practice for patients who manifested as progressive worsening or severe condition.

In addition, the blood purification system is one of the important life support systems for the treatment of critically ill patients and helps to remove proteins that are typically elevated during infections, which associated with a “cytokine storm” that occurs in some COVID-19 patients, leading to severe inflammation, rapidly progressive shock, respiratory failure, organ failure, and death.

As aforementioned, COVID-19 patients with CKD have higher risk of progression to critical illness or AKI. Moreover, critical condition or AKI may further aggravate the renal damage to an irreversible stage. Therefore, more attention should be paid to protection of renal function in the treatment of these patients to minimize drugs that may cause renal injury, such as antibiotics, contrast agents, HES, etc. If necessary, renal replacement therapy (RRT) should be initiated in a timely manner, particularly for those with strong inflammatory storm.

### ACEIs/ARBs in COVID-19 Patients with CKD

Angiotensin-converting enzyme inhibitors (ACEIs) and angiotensin receptor blockers (ARBs) are highly recommended medications for CKD patients especially with cardiovascular disease, hypertension, or diabetes. ACE2 served as not only the receptor for SARS-CoV-2 but also a key element in the protective arm of the renin-angiotensin system (RAS) [38]. RAS plays an important role in the pathophysiology of hypertension and cardiovascular and renal diseases [39]. Recently, a hypothesis suggested that patients taking ACEIs/ARBs may be at increased risk of severe disease outcomes during SARS-CoV-2 infection. ACE2 has also been suggested as a potential therapeutic target for SARS-CoV-2 [40].

To date, there is no high-quality evidence to support COVID-19 patients to adjust or discontinue ACEIs/ARBs treatment with exception for some special cases which concurred with complications including hyperkalemia, AKI, or hypotension. Therefore, many medical academic societies, including the European Society of Hypertension, International Society of Hypertension, and European Society of Cardiology [41–43], recommend continuing ACEIs/ARBs due to lack of evidence that taking ACEIs/ARBs drugs might increase susceptibility to SARS-CoV-2 infection nor is there sufficient evidence showing that ACEIs/ARBs may play a protective role in the treatment of COVID-19. While three studies recently reported the association between the use of ACEIs/ARBs and the risk of COVID-19 or in-hospital death [44–46], Mancia et al. [44] reported on a total of 6272 cases of patients infected with SARS-CoV-2 matched to 30,759 controls according to sex, age, and municipality of residence, and the use of ACEIs/ARBs did not show any association with COVID-19. The adjusted OR was 0.96 (95% CI 0.87–1.07) for ACEIs and 0.95 (95% CI 0.86–1.05) for ARBs. Another study by Reynolds et al. [45] also showed this association. In their study, in 12,594 patients who were tested for COVID-19, a total of 5894 (46.8%) were positive, and 1002 (17.0%) had severe illness, while neither ACEIs nor ARBs increased in the likelihood of a positive test for COVID-19 or in the risk of severe COVID-19 among patients who tested positive. An observational study by Mehra et al. [46] on 8910 patients with COVID-19 from 169 hospitals in Asia, Europe, and North America showed that coronary artery disease [10.2% vs. 5.2% among those without disease; OR 2.70 (95% CI 2.08–3.51)] was independently associated with an increased risk of in-hospital death, while no increased risk of in-hospital death was found to be associated with the use of ACEIs [2.1% vs. 6.1%; OR 0.33(95% CI 0.20–0.54)] or the use of ARBs [6.8% vs. 5.7%; OR 1.23 (95% CI 0.87–1.74)].

## Problems and Challenges

### The Exact Prevalence of CKD in COVID-19 Patients

The proportion of patients with COVID-19 combined with CKD is relatively lower (0–5.6%) [6, 8, 9, 11–14]. But the diagnosis of comorbidities mostly depends on self-report on admission with insufficient diagnostic testing which undermined the validity and accuracy [1]. For hypertension and diabetes, they are common comorbidities of COVID-19, and both diabetes and hypertension are high risk factors for CKD. Therefore, more relevant diagnostic testing is needed to elucidate the exact prevalence of CKD in COVID-19 patients, so as necessary renal function protection measures for potential CKD patients could be implemented.

### The Relationship Between SARS-CoV-2 and RAS Systems (Circulatory and Local)

Angiotensin-converting enzyme 2 (ACE2) is a key element in the protective arm of the renin-angiotensin system [47], and it also acts as a receptor for SARS-CoV-2. There are circulatory and local RAS systems. Moreover, there was no significant correlation between local renal RAS system and circulating RAS system. The circulating RAS system is based on angiotensinogen (AGT), which is mainly synthesized in the liver. There is another independent RAS system in the kidney that can self-sufficiently fulfill the biosynthetic process. AGT in the kidney mainly originates from proximal renal tubules [48–50]. In kidney tissues, SARS-CoV-2 infection was confirmed by immunofluorescence staining and transmission electron microscopy observations at autopsy in severe COVID-19 patients. SARS-CoV-2 mainly distributes in proximal tubular epithelium and podocytes which triggered some interesting questions: Does the use of ACEIs/ARBs lead to increased ACE2 expression and subsequently enhance viral entry? Does the interaction between viral S1 protein and ACE2 has an impact on the systematic or renal local RAS system? If yes, does the treatment of CKD patients need to be adjusted? Liu et al. reported that Ang II levels were higher in patients with COVID-19 compared with healthy controls, and it is correlated with lung injury (PaO2/FiO2). This was a small sample size study (including 12 patients), and unfortunately the authors did not mention way of blood sample collection and processing methods which could be critical in measuring the components of the RAS [51]. Further studies are still warranted for comprehensive understanding of the relationship between SARS-CoV-2 and RAS systems.

### Public Health Concern in CKD Patients During COVID-19 Outbreak

CKD is one of the major public health problems worldwide with a high prevalence and incidence in general population. HD patients need RRT in hospital. Social distancing and restrictions during the outbreak may substantially interfere the regular treatment of these patients. Monitoring of plasma concentration of immunosuppressive drugs may be inconvenient for renal transplant patients. Zhang et al. [52] reported that during the COVID-19 outbreak period, many problems in the management of Chinese children with CKD have been disclosed. In their study, they suggested that three-algorithm diagnosis and treatment system and “Internet + CKD health management” are desired for children with chronic diseases such as CKD, while the circumstances in adult CKD patients are also needed to explore.

### Long-Term Prognosis of COVID-19 Patients with CKD or AKI

Kidney injury was associated with higher mortality rates in COVID-19 patients and was an emerging concern to the clinicians [53], while the long-term prognosis of COVID-19 patients with renal injury is unknown. It was reported [21] that most COVID-19 patients with AKI will recover if their serum creatinine level is normal at admission. Pei et al. reported 251/333 (75.4%) patients who had abnormal urine dipstick tests or AKI. Patients with renal involvement had higher overall mortality compared with those without renal involvement [28/251 (11.2%) versus 1/82 (1.2%)]. The severity of pneumonia was the risk factor most commonly associated with lower odds of proteinuric or hematuric remission and recovery from AKI in stepwise multivariate binary logistic regression analyses [54]. We suggested that COVID-19 patients with renal injury during hospitalization should be regularly tested for routine urine and renal function after discharge in order to monitor patients for the development or progression of CKD after AKI [55]. If conditions permit, more sensitive indicators of renal injury such as urinary microalbumin, β-2 microglobulin, KIM-1, NGAL, TIMP-1*IGFBP7, and serum cysteine C should be tested. If the above indicators persist, abnormal or serum creatinine does not return to baseline levels, and consideration should be given to possible progression to CKD. If necessary, renal biopsy should be performed in time to make a definite diagnosis.
